# Real-world EUROCRINE^®^ registry data challenge the reliability of Bethesda cytopathology for thyroid surgery indication

**DOI:** 10.1515/iss-2021-0004

**Published:** 2021-08-17

**Authors:** Julia I. Staubitz, Alicia Poplawski, Felix Watzka, Thomas J. Musholt

**Affiliations:** Section of Endocrine Surgery, Department of General, Visceral and Transplantation Surgery, University Medical Center Mainz, Johannes Gutenberg-University Mainz, Mainz, Germany; Institute for Medical Biometry, Epidemiology and Informatics, University Medical Centre Mainz, Johannes Gutenberg-University Mainz, Mainz, Germany

**Keywords:** Bethesda reporting system, EUROCRINE, fine-needle aspiration cytology, thyroid cytopathology

## Abstract

**Objectives:**

Fine-needle aspiration cytology (FNAC) is recommended by international guidelines for the preoperative evaluation of suspicious thyroid nodules >1 cm. Despite robust evidence from endocrine centers demonstrating the key role of FNAC results for the indication of surgery, the method is not routinely used in European clinics. The database EUROCRINE^®^, which was introduced in 2015 with the scope of registering operations of the endocrine system, allows for a large-scale analysis of the current service reality in Europe concerning FNAC use and associated accuracy.

**Methods:**

Operations performed to “exclude malignancy”, registered from January 2015 to December 2018 in EUROCRINE^®^, were analyzed. Parameters of accuracy were calculated for FNAC. FNAC results were considered “test positive” in the case of Bethesda category IV, V, and VI, since these categories usually prompt surgical interventions in European centers for thyroid surgery. Bethesda category II and III were considered “test negative”.

**Results:**

Of 8,791 operations, 5,780 had preoperative FNAC (65.7%). The overall malignancy rate was 28.3% (2,488/8,791). Malignancy rates were 68.8% for Bethesda VI, 69.9% for Bethesda V, 32.6% for Bethesda IV, 28.2% for III, 20.2% for Bethesda II, and 24.5% for Bethesda I. After exclusion of papillary microcarcinomas (PTMCs), the sensitivity of FNAC was 71.7% and specificity 43.5%, the positive predictive value was 29.1% and the negative predictive value 82.7%.

**Conclusions:**

Although the indication to “exclude malignancy” was the predominant reason that prompted thyroid resection in the present cohort, FNAC was only used in about 65.7% of cases. When performed, FNAC was associated with unexpectedly low accuracy. Interestingly, in Bethesda II, 20.2% of malignant entities were present (13.3% after the exclusion of PTMCs).

## Introduction

The Bethesda System for Reporting Thyroid Cytopathology was established in 2009 as a category-based system for the analysis of thyroid fine-needle aspiration cytology (FNAC) specimens and was revised in 2017 [1], [2], [3]. Six categories were defined, which are denominated as follows: Bethesda I = nondiagnostic or unsatisfactory, Bethesda II = benign, Bethesda III = atypia of undetermined significance or follicular lesion of undetermined significance, Bethesda IV = follicular neoplasm or suspicious for a follicular neoplasm, Bethesda V = suspicious for malignancy and Bethesda VI = malignant. Each category is associated with a specific cancer risk, described to range from 5 to 10% for Bethesda I and from 0 to 3% for Bethesda II [1]. For Bethesda III, the risk of malignancy was reported to range from 10 to 30%, for Bethesda IV from 25 to 40%, for Bethesda V from 50 to 75%, and for Bethesda VI from 97–99% [1].

However, the introduction of the novel entity of noninvasive follicular thyroid neoplasia with papillary nuclear features (NIFTP) by the WHO in 2017 [4] led to a change in the attributed cancer risk for Bethesda categories III to VI [1]. Based on the redefinition of an entity (previously defined as a papillary thyroid carcinoma [PTC]) as a borderline tumor with debatable malignant potential, the risk of malignancy in Bethesda categories III–VI was lowered, as published by Cibas and Ali in 2017 [1].

The value of FNAC was strongly emphasized by the “Guidelines for Adult Patients with Thyroid Nodules and Differentiated Thyroid Cancer” published by the American Thyroid Association (ATA) in 2015: FNAC and the assignment to Bethesda categories holds a central position in the recommended algorithm for the evaluation of sonographically suspicious thyroid nodules and should be performed prior to surgical excision [5].

The European database EUROCRINE^®^, which was introduced in 2015, allows for a large-scale analysis of the current service reality in specialized centers for endocrine surgery with regard to the actual use of FNAC and corresponding malignancy rates behind Bethesda categories in operations, performed with the primary intention to “exclude malignancy”. The clinics participating in EUROCRINE^®^ are primarily centers that actively took the decision to share their perioperative data with other European centers in order to optimize the surgical care in the field of endocrine diseases. As a result, epidemiological analyses of the registry are not representative of general health care but reflect the care provided in clinics devoted to endocrine surgery.

## Methods

### Operations

All thyroid operations, which were entered in the European database EUROCRINE^®^ for the registered main indication “excluding malignancy” from January 2015 to December 2018, were included in the study (data of 99 hospitals from 11 different countries). Pre, intra, and postoperative parameters were assessed, including results of FNAC analysis and the rate of completion thyroidectomies (i.e., redo surgery, performed within 6 months following an inadequate primary operation to facilitate radioiodine therapy).

### EUROCRINE^®^ registry

The European registry EUROCRINE^®^ has been available since 2015 for hospitals with a focus on endocrine surgery. The project was initiated in 2013 as part of the “Health Program” of the European Union. Since 2018, the EUROCRINE^®^ Society, based in Vienna, Austria, has been registered as a nonprofit organization. EUROCRINE^®^ is directed by a steering committee consisting of representatives of the national surgical societies and the European Society of Endocrine Surgery [6]. The objectives of the EUROCRINE^®^ registry are the reduction of morbidity and mortality due to diseases of the endocrine system, which is aimed for by an international comparison of the applied therapy strategies [6, 7]. Operations registered with EUROCRINE^®^ refer to the thyroid, parathyroid, neuroendocrine tumors of the digestive tract, or extra-adrenal paraganglia. In addition to the systematic documentation of endocrine surgery, the registry also offers the possibility of recording indications, preoperative diagnostics, perioperative management, and extensive postoperative follow-up examinations. Data completeness and validity are reviewed by audits as well as internal algorithms for plausibility checks.

### Statistical analysis

Data were documented and described using Microsoft Excel (Microsoft Corporation, Redmond, USA). Statistics were performed using R (v. 3.6.2, R Core Team (2019). R: A language and environment for statistical computing. R Foundation for Statistical Computing, Vienna, Austria). Comparisons between groups were performed with Kruskal–Wallis test, Chi-squared test, Fisher’s exact test, or Fisher’s exact test with the simulated p-value. Categorical variables are presented as numbers and percentages, and continuous variables as median with range. A p-value of <0.05 from a two-sided test was considered statistically significant. For the analysis of the parameters of the accuracy of FNAC, only conclusive FNAC results (=Bethesda categories II–VI) were included (exclusion of Bethesda category I). FNAC results were considered “test positive” in the case of Bethesda category IV, V, and VI, since these categories usually prompt surgical interventions in European centers for thyroid surgery. Bethesda category II and III were considered “test negative”. Defined as “test positive” in gold standard histology were malignant tumors. An additional calculation of the parameters of the accuracy of FNAC was performed analogously, for which papillary microcarcinoma (PTMC) was defined as “test negative” in the gold standard. Furthermore, calculations for parameters of accuracy were performed, after the exclusion of different entities (FTC, medullary thyroid carcinoma, and metastatic (M1) carcinomas) from the total number of analyzed carcinomas. Funnel plots were created to visualize the rate of FNAC, the rate of carcinomas, and the rate of completion of thyroidectomy in the different EUROCRINE^®^ hospitals (Microsoft Excel, Microsoft Corporation, Redmond, USA).

## Results

**Figure 1:**
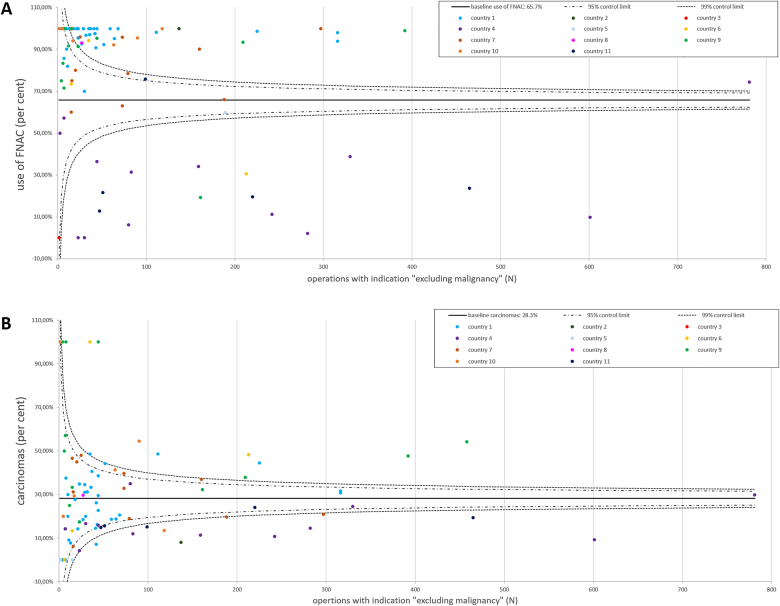
EUROCRINE^®^ 2015–2018: use of FNAC and histologically confirmed carcinomas in operations with the intention to “exclude malignancy”. (A) The relative use of FNAC in the participating 99 EUROCRINE^®^ hospitals from 11 countries is illustrated. Each EUROCRINE^®^ hospital is indicated as a dot, according to the total number of operations performed in the specific center. Hospitals belonging to one country are indicated by the same color. The baseline use of FNAC was 65.7%. (B) The percentage of carcinomas among all operations to “exclude malignancy” is presented for the participating 99 hospitals from the included 11 EUROCRINE^®^ countries. Each EUROCRINE^®^ hospital is indicated as a dot, according to the total number of operations performed in the specific center. Hospitals belonging to one country are indicated by the same color. The baseline of carcinomas in operations performed to “exclude malignancy” was 28.3%.

**Figure 2:**
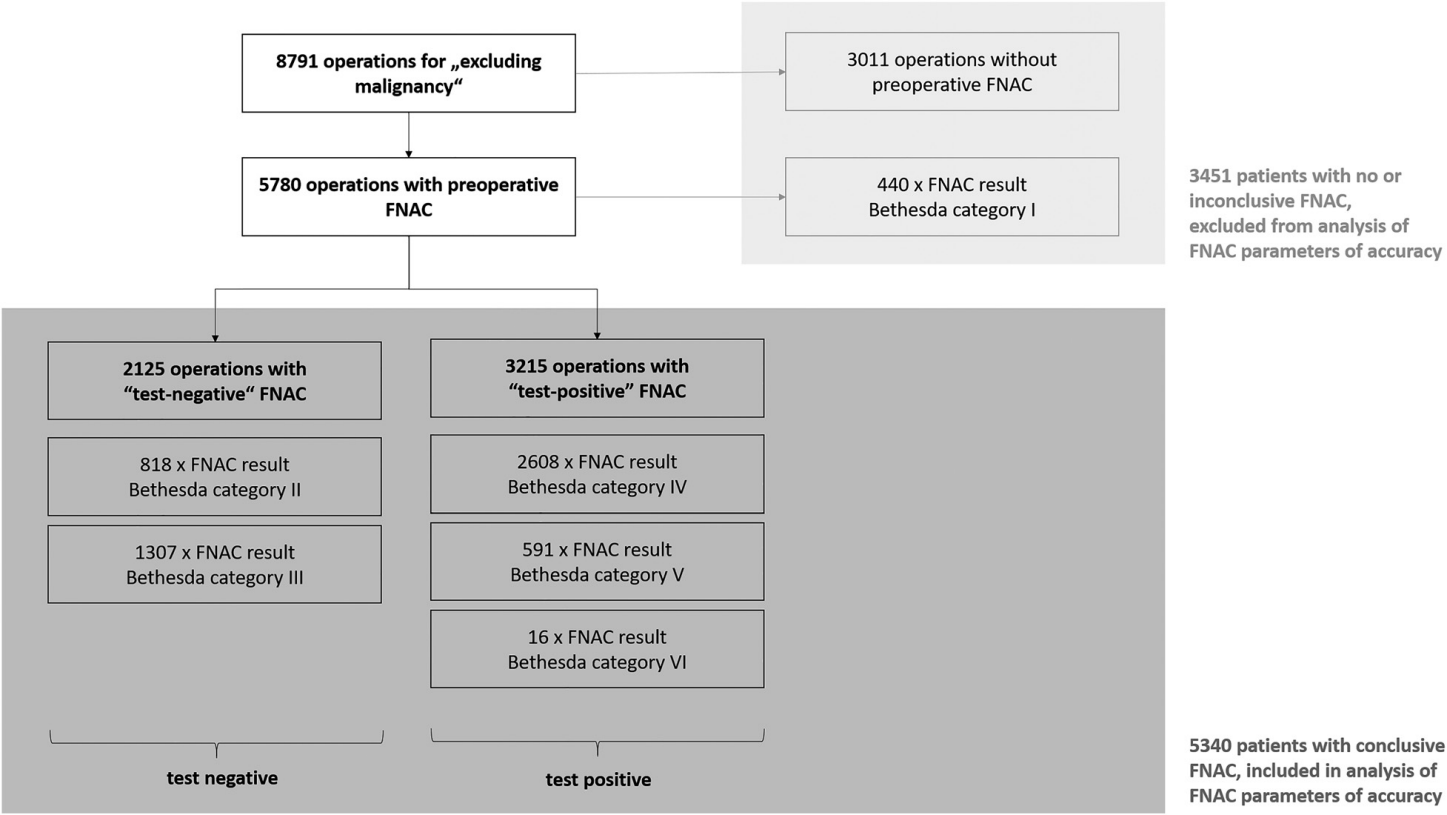
Patient flow – basis for analysis of accuracy and impact of preoperative FNAC. In the analyzed cohort, 5,340 patients had FNAC with conclusive results. Defined as “test positive” in preoperative FNAC were results with Bethesda category IV–VI since these categories usually prompt surgical interventions in European centers for thyroid surgery. Bethesda category II and III were considered “test negative”. Bethesda category I, which includes nondiagnostic and therefore inconclusive results, and operations without preoperative FNAC were excluded from the analysis of FNAC parameters of accuracy.

Of a total of 8,791 operations, which were registered in EUROCRINE^®^ in the study period, 65.7% (5,780/8,791) underwent preoperative FNAC (Figure 1A, Table 1). FNAC was ultrasound guided in 87.9% (5,079/5,780). Of all FNAC analyses, 7.6% were not conclusive (440/5,780, Figure 2, Table 2). Whereas of the inconclusive FNAC, 86.1% (379/440) were ultrasound guided, 88.0% (4,700/5,340) of the conclusive cytologies were obtained with ultrasound guidance. The malignancy rate of the present cohort was 28.3% (2,488/8,791, Figure 1B).

**Table 1:** Pre, intra, and postoperative parameters according to Bethesda category.

Bethesda categories [1]	I	II	III	IV	V	VI	p-Value
Total operations, n	440	818	1,307	2,608	591	16	–
Patient age (median, range)	53, 11–84	52, 4–87	53, 12–89	52, 11–91	53, 10–90	57, 25–78	^a^0.256
Patient sex male	116, 26.4	177, 21.6	267, 20.4	546, 20.9	133, 22.5	3, 18.8	^b^0.154
Previous thyroid operation, n	87, 19.8	139, 17.0	329, 25.2	496, 19.0	158, 26.7	2, 12.5	^b^<0.001
Thyroid procedure							^b^<0.001
Lobectomy, n, %^d^	275, 62.5	467, 57.1	871, 66.7	1,920, 73.6	194, 32.8	5, 31.5	
Thyroidectomy, n, %^d^	151, 34.3	330, 40.3	403, 30.8	640, 24.5	389, 65.8	11, 68.8	
Isthmus resection, n, %^d^	4, 0.9	7, 0.9	24, 1.8	32, 1.3	4, 0.7	0, 0	
Other operation, n, %^d^	10, 2.3	14, 1.7	9, 0.7	16, 0.6	4, 0.7	0, 0	
Lymphadenectomy, n	56, 12.7	72, 8.8	229, 17.5	340, 13.0	305, 51.6	7, 43.8	^b^<0.001
Malignant histology, n, %^d^	108, 24.5	165, 20.2	369, 28.2	851, 32.6	413, 69.9	11, 68.8	^c^<0.001
Malignancy rate after exclusion of PTMC, n, %^d^	71, 16.1	109, 13.3	259, 19.8	604, 23.2	320, 54,1	10, 62.5	^c^<0.001
Carcinomas (n), of these:	108	165	369	851	413	11	^c^<0.001
– PTMC, without concomitant carcinoma, n, %^e^	37, 34.3	56, 33.9	110, 29.8	247, 29.0	93, 22.5	1, 9.1	
– PTC, >1 cm diameter, n, %^e^	48, 44.4	77, 46.7	184, 49.9	354, 41.6	272, 65.9	8, 72.7	
– FTC, n, %^e^	15, 13.9	18, 10.9	63, 17.1	194, 22.8	31, 7.5	1, 9.1	
– MTC, n, %^e^	4, 3.7	11, 6.7	6, 1.6	10, 1.2	11, 2.7	0, 0	
– PDTC, n, %^e^	0, 0	2, 1.2	3, 0.8	17, 2.0	4, 1.0	0, 0	
– ATC, n, %^e^	3, 2.7	0, 0	1, 0.3	4, 0.5	1, 0.2	0, 0	
– Other carcinomas, n, %^e^	1, 1.0	1, 0.6	2, 0.5	25, 2.9	1, 0.2	1, 9.1	
Completion thyroidectomy, n, %^e^	11, 10.2	9, 5.5	58, 15.7	144, 16.9	26, 6.3	1, 9.0	^c^<0.001

^a^Kruskal–Wallis test. ^b^Chi-squared test. ^c^Fisher’s exact test with simulated p-values. ^d^Percentage referred to a total number of operations. ^e^Percentage referred to total numbers of carcinomas. PTMC, papillary microcarcinoma; PTC, papillary thyroid carcinoma; FTC, follicular thyroid carcinoma; MTC, medullary thyroid carcinoma; PDTC, poorly differentiated thyroid carcinoma; ATC, anaplastic thyroid carcinoma.

**Table 2:** Sensitivity of FNAC after exclusion of different entities from the analysis.

	Excluded entity	Sensitivity, %	Specificity, %	Positive predictive value, %	Negative predictive value, %
1	None	70.5	45.1	39.7	74.9
2	Papillary microcarcinoma (PTMC)	71.7	43.5	29.1	82.7
3	Follicular thyroid carcinoma (FTC)	69.8	45.1	35.1	77.8
4	Medullary thyroid carcinoma (MTC)	70.8	45.1	39.3	75.5
5	Carcinomas with distant metastases (M1)	70.4	45.1	39.5	75.0
6	Entities 2–5	71.7	43.5	23.0	86.8

Based on the malignancy rates presented in Table 1, FNAC sensitivity was 70.5%. The specificity of FNAC was 45.1%. The positive predictive value (PPV) was 39.7% and the negative predictive value (NPV) was 74.9% (Table 2). The highest proportions of malignant entities were expectedly present in Bethesda V and VI (69.9 and 68.8%, respectively). Bethesda categories III and IV had similarly high results of 28.2 and 32.6%. The lowest malignancy rate was present in Bethesda II: 20.2%. The majority of carcinomas in all categories were, as expected, PTC (Table 1). The highest frequency of follicular thyroid carcinomas (FTC) was registered in categories IV > III > I (22.8 > 17.1>13.9% of carcinomas within each category, Table 1). However, PTMCs were present in all Bethesda categories. Assuming that the PTMCs were not the target of FNAC, a second calculation of the parameters of accuracy was performed, which provided a sensitivity of 71.7%, a specificity of 43.5%, a PPV of 29.1%, and an NPV of 82.7%. Malignancy rates of 16.1% in Bethesda I, 13.3% for Bethesda II, 19.8% for Bethesda III, 23.2% for category IV, 54.1% for Bethesda V, and 62.5% for Bethesda VI were calculated after the exclusion of PTMCs. Parameters of accuracy, calculated additionally after the exclusion of different entities, which might have compromised FNAC sensitivity (as FTC, MTC, and preoperatively known metastatic carcinomas) are presented in Table 2.

**Figure 3:**
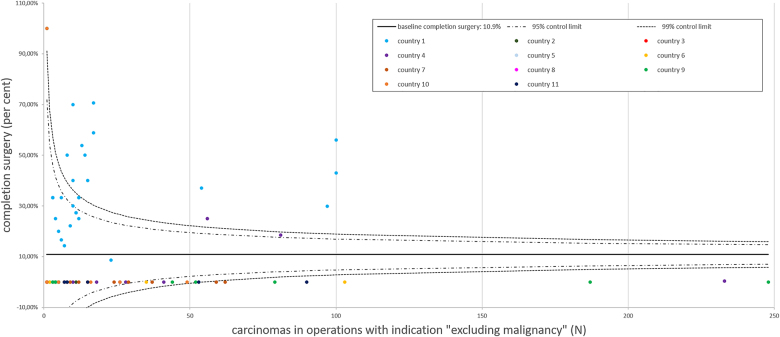
EUROCRINE 2015–2018: completion surgery for carcinoma. The baseline for completion surgery in relation to carcinomas operated at the different EUROCRINE^®^ hospitals was 10.9%. A reporting bias is possible: completion surgery cases for thyroid carcinoma in the same patients, which were registered with different patient IDs, were not traceable within the registry. Each EUROCRINE^®^ hospital is indicated as a dot, according to the total number of carcinomas operated by the specific center. Hospitals belonging to one country are indicated by the same color.

The overall rate of completion thyroidectomy of carcinomas in the present cohort was 10.9% (270/2,488, Figure 3). In the subgroup with conclusive FNAC (referred to the primary operation, which required completion thyroidectomy in the further course), 1,809 carcinomas were present (33.9%, 1,809/5,340). Of these, 238 required completion surgery (13.2%, 238/1,809). In comparison, the subgroup with absent or inconclusive FNAC, which preceded the initial operation, harbored 679 carcinomas (19.7%, 679/3,451) and completion surgery was performed in 32 patients (4.7%, 32/679, p < 0.001).

## Discussion

In the present cohort, FNAC was performed in 65.7% of patients with thyroid nodules that were referred to surgery for the main indication “excluding malignancy” and in 60.7% of all patients, conclusive FNAC results (Bethesda II–VI) were available. This result illustrates that the current service reality in European centers of endocrine surgery is not in line with the recommendations for the surgical approach published by the ATA, which are essentially based on the result of FNAC for decision-making toward an operation [5]. However, comprehensible reasons to abstain from FNAC are elevated basal calcitonin values, which indicate the presence of medullary thyroid carcinoma, the clear indication of malignant diagnoses by radiological imaging (e.g., with cervical lymph node metastases), small nodule size of less than 1 cm in diameter or the fact that the patient refuses this preoperative diagnostic option. In cases, for which the intended surgical strategy is thyroidectomy in any case, and therefore a sufficient therapy for most thyroid carcinomas, surgeons might also have abstained from preoperative FNAC.

Surprisingly, in the cohort of operations with the main intention “to exclude malignancy”, there were 16 operations, which were preceded by FNAC Bethesda category VI. In these cases, incorrect coding might be the underlying reason, as the EUROCRINE^®^ category “operation for malignancy” would have been more suitable. Yet, one of these 16 operations required completion surgery, which implies, that the result of malignant FNAC indication did not prompt an adequately radical surgical intervention in the first place. However, the resection extent in the present cohort significantly differed between Bethesda V/VI and Bethesda I–IV. In the case of Bethesda V and VI, predominantly thyroidectomies were performed as well as lymph node dissections. In the remaining categories (Bethesda I–IV), mainly lobectomies were performed and lymph node dissections were relatively rare. This illustrates that the result of Bethesda V/VI in preoperative FNAC prompted oncological resections, whereas numerous procedures following the result of Bethesda I–IV may have partly been diagnostic lobectomies.

A drawback of the present study is that it was not documented, whether the target lesions of FNAC were the actual carcinomas detected in final histology. Referring to the recommendations of the ATA and the Thyroid Section of the German association of endocrinology [5, 8], which do not recommend FNAC in thyroid nodules <1 cm in size, it can be assumed that the majority of cases of papillary thyroid microcarcinomas on final histology were diagnosed incidentally. However, also the additionally performed calculation of the parameters of accuracy, which excluded these potentially concomitant PTMC cases, showed debatable parameters of accuracy. Underlying was less evident differences between the malignancy rates in the different Bethesda categories than reported in the literature [1]. Whereas in Bethesda II, 0–3% carcinomas were expected according to the literature [1, 9], a rate of 20.2 and 13.3% (after the exclusion of PTMCs, respectively) was documented. A similar rate of malignancy of 12.7% in Bethesda II was reported by Inabnet et al., who analyzed a cohort of 21,746 patients undergoing FNAC and subsequent thyroid surgery [10]. According to the ATA 2015 guidelines, following benign cytology, diagnostic thyroid surgery is not recommended (recommendation 11) [5]. However, in the present cohort, the operating surgeons decided for an operation to exclude malignancy, despite benign cytology results in a total of 818 cases. In this study, the malignancy rate within Bethesda III was expectedly between 10 and 30%, as reported in the literature: namely 28.2 and 19.8% (after the exclusion of PTMCs) were registered. However, the malignancy rates in Bethesda categories IV, V, and VI were below the rates described in the literature, which is reflected by the low sensitivity of FNAC, which was 70.5 and 71.7% after the exclusion of PTMC.

Also, in other studies with different approaches to assessing FNAC parameters of accuracy, surprisingly low sensitivities were documented [10, 11]. However, the method of calculation for sensitivity and specificity, which was chosen for this analysis, also had a defining impact on the parameters of accuracy. Bethesda IV, V, and VI were set as “test positive” since these categories usually prompt thyroid resections in European endocrine surgery centers. A tighter choice of only categories V and VI as “test positive” results would have allowed for stronger parameters of accuracy, but numerous FTCs (highest number present in category IV; namely 194 carcinomas) would have been missed. Therefore, this calculation consciously accepts false-positive results. Finally, the restriction to operations performed with the main intention to “exclude malignancy”, as encoded by the operating surgeon, bears a selection bias. Yet, it should also be acknowledged, that the indication for surgery was frequently based on more than one reason, e.g., a suspicious nodule in large multinodular goiter with compression symptoms, while this analysis considered only the leading indication. Furthermore, the operations which followed FNAC results with Bethesda category VI might represent coding errors within this category (more suitable for EUROCRINE^®^ category: operation for “malignancy”). However, when assuming the ideally prevailing malignancy rates published by Ali and Cibas [1], following the applied model of analysis for the total number of cytologies performed in the present cohort, a sensitivity of 78.3%, a specificity of 49.9%, a PPV of 46.7 and NPV of 80.4% could have been expected. However, not even an additionally performed exclusion of histological entities, which might have led to a weaker accuracy (FTC, PTMC, MTC, and M1 carcinoma), was able to demonstrate this favorable result: sensitivity was 71.7%, specificity was 43.5%, PPV was 23.0%, and NPV was 86.8% (Table 2).

Another interesting observation was the difference in the necessity of completion surgery for oncological reasons in patients, who underwent FNAC with the conclusive result, in comparison to patients without or with inconclusive FNAC. The rates of completion surgery were 13.2% for the subgroup with conclusive FNAC and 4.7% for the subgroup without or with inconclusive FNAC. This is against the expectation that preoperative FNAC assessment improves surgical management, and thereby avoids secondary surgery. However, indications for surgery in the present cohort were established after sonographic evaluation, taking into consideration clinical development, radioiodine scans, and molecular assessment as well as results provided by FNAC. FNAC has to be seen as a tool in a complex framework for the decision-making toward an operation. Especially a combination of systematic ultrasound evaluation of thyroid nodules according to TI-RADS was shown to be of utility [12]. In the future, risk-adapted surgery might additionally be based on molecular characteristics, in addition to classical cytological criteria. The discovery of *BRAFV600E* as a driver mutation, which represents a pathognomonic alteration for PTC, led to an increase of the sensitivity of preoperative FNAC of about 10%, if the additional molecular analysis is taken into consideration [13], [14], [15], [16]. Moreover, the detection of *BRAF* mutation can rule out the diagnosis of NIFTP, which knowingly complicates the cytological diagnosis [17]. Furthermore, the association between *TERT* promoter and *BRAFV600E* mutations creates a unique mechanism for the amplification of *TERT* expression, which results in higher tumor aggressiveness [18, 19], which may justify a more radical surgical approach.

Finally, the overall frequency of malignant disease of 26% in the described cohort of patients operated to “exclude malignancy” raises the question if the preoperative evaluation of thyroid nodules must be improved in order to avoid unnecessary resections. On the other hand, it is generally accepted that risk of malignancy of more than 5% justifies surgery. Moreover, large follicular adenomas, oncocytic adenomas, and other neoplasias with uncertain malignant potential may represent pre-cancerous lesions that add to the benefit of these operations.

## Conclusion

In the centers of endocrine surgery, which contributed their data to the database EUROCRINE^®^ in the period from 2015 to 2018, FNAC was preoperatively performed in 65.7% of patients, who were referred to surgery to “exclude malignancy”. Unexpectedly low parameters of accuracy were documented for FNAC. Against the recommendation not to perform diagnostic thyroid surgery following benign FNAC results, 818 patients with Bethesda II thyroid nodules underwent surgery to exclude malignancy, which was confirmed in 20.2% of these cases (13.3% after the exclusion of PTMCs). Therefore, future guidelines should acknowledge the limitations of FNAC especially with regard to Bethesda category II. Finally, the use of FNAC did not lead to a reduced rate of completion thyroidectomy in the analyzed cohort.

## Supplementary Material

Supplementary Material
