# Supplementary material for: Real-world EUROCRINE® registry data challenge the reliability of Bethesda cytopathology for thyroid surgery indication
Source: Innov Surg Sci. 2021 Aug 17;7(3-4):99–106. doi: 10.1515/iss-2021-0004 (PMC9742262; doi:10.1515/iss-2021-0004)
Supplement: Supplementary file 1 — Supplementary Material [file j_iss-2021-0004_suppl.pdf]

Reviewer Assessment

J Staubitz, et al.: Real-world EUROCRINE® registry data challenge the reliability of Bethesda cytopathology for thyroid surgery indication

Reviewers' Comments to Original Submission

Reviewer 1: anonymous

Date received: 16-Feb-2021

Reviewer recommendation: **Reject for the following reasons**

Reviewer overall scoring: **Unacceptable**

Assessment Form scores: 5 = High/Yes; 3 = Medium/Adequate; 1 = Low

Is the subject area appropriate for the journal	5				
Does the title clearly reflect the paper's content?			3		
Does the abstract clearly reflect the paper's content			3		
Do the keywords clearly reflect the paper's content?			3		
Does the introduction present the problem clearly?				2	
Are the results/ conclusions justified?					1
How comprehensive and up-to-date is the subject matter presented?					1
How adequate is the data presentation?				2	
Are units and terminology used correctly?	5				
Is the number of cases adequate?					1
Are the experimental methods/ clinical studies adequate?					1
Is the length appropriate in relation to the content?	5				
Does the reader get new insights from the article?					1
Please rate the practical significance.					1
Please rate the accuracy of methods.					1
Please rate the statistical evaluation and quality control.			3		
Please rate the appropriateness of the figures and tables.			3		
Please rate the appropriateness of the references.	5				
Please evaluate the writing style and use of language.		4			
Please judge the overall scientific quality of the manuscript.					1
Are the methods used worthy of reproduction in greater deal?	No				
Would you be willing to review a revision of this manuscript?	Yes				

Comments to author: Retrospective study aiming to correlate results of fine needle aspiration biopsy (FNAB) with surgical pathology after lobectomy or total thyroidectomy for thyroid nodules classified by the TBSRTC (The Bethesda System for Reporting Thyroid Cytopathology). 8791 surgeries were included in the multiinstitutional EUROCRINE database with 5079 of them having had preop FNAB. Malignancy rates after exclusion of papillary microcancer (PTMC) were reported as to be 16.1%

for Bethesda class 1 (B1), 13.3% (B2), 19.8% (B3), 23.2% (B4) 54.1% (B5), and 62.5% for B6. With PTMC included the results for B1-B6 were 24.5%; 20.2%; 28.2%; 32.6%; 69.9%; and 68.8%.

1. M&M do not describe in- and exclusion criteria, in particular nodularity (single vs. multiple) was not defined.
2. M&M do not describe method of FNAB including method and use of ultrasound for targeting thyroid nodule(s).
3. Whether FNAB was defined exclusively as same-nodule histologic pathology was not reported.
4. Results: compared with results from the literature (e.g. Ali SZ, Cibas Es, The Bethesda System for Thyroid Cytopathoogy, 2009; malignancy rates of B1 to B6 1-4%; 0-3%; 5-15%; 15-30%; 60-75%; and 97-99%) data of this study are hardly to understand. 16.1% and 24.5% for Bethesda 1, defined as "nondiagnostic or unsatisfactory", does not meet the understanding and consensus of TBSRTC, as of B2 ("benign")(13.3%/20.2% vs. 0-3%), B3 ("atypia of undetermined significance or follicular lesion of undetermined significance") (19.8%/28.2% vs. 5-15%), and B6 ("malignant") (62.5%/68.8% vs. 97-99%). Due to inadequate definitions of target lesions, FNAB method, and consistency of TBSRTC it is rather impossible to clarify the differences between EUROCRINE data and international literature.

Reviewer 2: Geffcken, Christine

Date received: 18-Mar-2021
Reviewer recommendation: **Accept in present form**
Reviewer overall scoring: **Excellent**

Assessment Form scores: 5 = High/Yes; 3 = Medium/Adequate; 1 = Low

Is the subject area appropriate for the journal	5				
Does the title clearly reflect the paper's content?	5				
Does the abstract clearly reflect the paper's content	5				
Do the keywords clearly reflect the paper's content?	5				
Does the introduction present the problem clearly?	5				
Are the results/ conclusions justified?	5				
How comprehensive and up-to-date is the subject matter presented?	5				
How adequate is the data presentation?	5				
Are units and terminology used correctly?	5				
Is the number of cases adequate?	5				
Are the experimental methods/ clinical studies adequate?	5				
Is the length appropriate in relation to the content?	5				
Does the reader get new insights from the article?	5				
Please rate the practical significance.	5				
Please rate the accuracy of methods.	5				
Please rate the statistical evaluation and quality control.	5				
Please rate the appropriateness of the figures and tables.	5				
Please rate the appropriateness of the references.	5				
Please evaluate the writing style and use of language.		4			
Please judge the overall scientific quality of the manuscript.	5				
Are the methods used worthy of reproduction in greater deal?	Yes				
Would you be willing to review a revision of this manuscript?	Yes				

Comments to author: The filler word "however" is used a lot of times in the paper (I counted 8 times in the discussion). I recommend to change it partially or omit it.

Reviewer 3: Scheuba, Christian

Date received: 19-May-2021
Reviewer recommendation: **Accept in present form**
Reviewer overall scoring: **Excellent**

Assessment Form scores: 5 = High/Yes; 3 = Medium/Adequate; 1 = Low

Is the subject area appropriate for the journal	5				
Does the title clearly reflect the paper's content?	5				
Does the abstract clearly reflect the paper's content	5				
Do the keywords clearly reflect the paper's content?	5				
Does the introduction present the problem clearly?	5				
Are the results/ conclusions justified?	5				
How comprehensive and up-to-date is the subject matter presented?	5				
How adequate is the data presentation?	5				
Are units and terminology used correctly?	5				
Is the number of cases adequate?	5				
Are the experimental methods/ clinical studies adequate?	5				
Is the length appropriate in relation to the content?		4			
Does the reader get new insights from the article?	5				
Please rate the practical significance.	5				
Please rate the accuracy of methods.	5				
Please rate the statistical evaluation and quality control.	5				
Please rate the appropriateness of the figures and tables.		4			
Please rate the appropriateness of the references.	5				
Please evaluate the writing style and use of language.		4			
Please judge the overall scientific quality of the manuscript.	5				
Are the methods used worthy of reproduction in greater deal?					
Would you be willing to review a revision of this manuscript?	Yes				

Comments to author: Excellent work

Comments by the Editor-in-Chief to Submission

Considering all conflicting comments and recommendations by the three reviewers, I deem the contribution suitable for publication in our journal.
